# Antitumoral and Immunomodulatory Effect of *Mahonia aquifolium* Extracts

**DOI:** 10.1155/2019/6439021

**Published:** 2019-12-14

**Authors:** Andra Diana Andreicuț, Eva Fischer-Fodor, Alina Elena Pârvu, Adrian Bogdan Ţigu, Mihai Cenariu, Marcel Pârvu, Florinela Adriana Cătoi, Alexandru Irimie

**Affiliations:** ^1^Pathophysiology, Faculty of Medicine, University of Medicine and Pharmacy Iuliu Hațieganu, RO-400012 Cluj-Napoca, Romania; ^2^Medfuture Research Center for Advanced Medicine, University of Medicine and Pharmacy Iuliu Hațieganu, RO-400012 Cluj-Napoca, Romania; ^3^Tumor Biology Department, The Oncology Institute I. Chiricuță, RO--, 400015 Cluj-Napoca, Romania; ^4^Faculty of Biology and Geology, Babeș-Bolyai University, RO-400015 Cluj-Napoca, Romania; ^5^Biotechnology Research Center, University of Agricultural Science and Veterinary Medicine, Cluj-Napoca, Romania; ^6^Oncology, Faculty of Medicine, University of Medicine and Pharmacy Iuliu Hațieganu, RO-400012 Cluj-Napoca, Romania

## Abstract

The prodrug potential of *Mahonia aquifolium*, a plant used for centuries in traditional medicine, recently gained visibility in the literature, and the activity of several active compounds isolated from its extracts was studied on biologic systems *in vitro* and *in vivo*. Whereas the antioxidative and antitumor activities of *M. aquifolium*-derived compounds were studied at some extent, there are very few data about their outcome on the immune system and tumor cells. To elucidate the *M. aquifolium* potential immunomodulatory and antiproliferative effects, the bark, leaf, flower, green fruit, and ripe fruit extracts from the plant were tested on peripheral blood mononuclear cells and tumor cells. The extracts exert fine-tuned control on the immune response, by modulating the CD25 lymphocyte activation pathway, the interleukin-10 signaling, and the tumor necrosis-alpha secretion in four distinct human peripheral blood mononuclear cell (PBMC) subpopulations. *M. aquifolium* extracts exhibit a moderate cytotoxicity and changes in the signaling pathways linked to cell adhesion, proliferation, migration, and apoptosis of the tumor cells. These results open perspectives to further investigation of the *M. aquifolium* extract prodrug potential.

## 1. Introduction

In the tumor microenvironment (TME), tumor intrinsic factors and tumor extrinsic factors work together to induce immunosuppression. The composition of TME depends on the cancer types and disease stages. The tumor cells chronically secrete tumor intrinsic factors. Some of them induce reduction of the immune effector cell activity and promote immune evasion by decreasing the expression of antigen-presenting molecules and by expressing neoantigens. In the same time, the tumor cells use autocrine or paracrine signals in order to stimulate the expression of immune checkpoints (ICs) [1, 2] on immune cells and to upregulate immunosuppressive cell recruitment and activation [3]. The tumor cells also secrete cytokines and growth factors that promote tumor growth, angiogenesis, and metastasis [4].

The tumor extrinsic factors are provided by immune and nonimmune cells [4]. The immune cells of the TME are T cells, B cells, macrophages, monocytes [5], dendritic cells, and NK cells [6], and an efficient antitumor immune response implies both helper CD4+ and effector CD8+ T cells activated in proximity to each other [7]. T regulatory cells (Tregs) are a subset of CD4+ T cells, with immunosuppressive activity by inhibiting cytotoxic CD8+ T cells and effector CD4+ T cell activation, via consumption of IL-2, release of TGF-*β* and IL-10, and IC expression upregulation [4, 8]. B regulatory cells (Bregs), a subset of B cells, and tumor-associated macrophages (TAMs) reduce the activity of cytotoxic CD8+ and CD4+ T effector cells by releasing anti-inflammatory cytokines like IL-10 and by expressing coinhibitory molecules [9–11]. TAMs also stimulate tumor growth and metastasis by secreting matrix metalloproteinases (MMPs) and proangiogenic factors, like VEGF [10]. Other tumor-associated immune cells are myeloid-derived suppressor cells (MDSCs) [12], a heterogeneous population of myeloid cell precursors that can suppress cytotoxic CD8+ T cell; tumor-associated mast cells (TAMCs), with a controversial immunosuppressive role; and tumor-associated dendritic cells (TADCs), which can inhibit cytotoxic CD8+ T cells by expressing inhibitory molecules and releasing IL-10 and TGF-*β* [13].

From the TME nonimmune cells, the most important are cancer-associated fibroblasts (CAFs) and tumor endothelial cells (TECs) [4]. As a response to TME hypoxia and tumor cell intrinsic factor release, normal resident fibroblasts are converted to CAFs [14, 15]. They stimulate tumor growth, invasion and metastasis, MMPs, angiogenesis, and CD8+ T cell apoptosis [4]. TECs are different from the normal epithelial cells due to the morphological abnormalities and induce angiogenesis with new vessel formation that allows tumor cell metastasis. Moreover, active TECs release their own growth and angiogenic factors, further stimulating neighboring tumor cell growth [16, 17].

The cells of the immune system should stop the tumor growth and the progression by recognition and removal of the malignant cells [18]. Instead, it was found that the result of the interaction between immune and nonimmune cells in the TME is tumor-mediated immunosuppression [4, 19]. Furthermore, the tumor-mediated immunosuppression may also reduce cancer therapy efficiency and may induce resistance to therapy.

The genus Mahonia Nuttall has about 70 species, and it is the second largest genus in the Berberidaceae family. Mahonia plants are native to Eastern Asia, North America, and Central America [20] and have been widely used in traditional medicine for centuries. It was shown that Mahonia species have antioxidant, anti-inflammatory [21, 22], antifungal, antimicrobial [23], antiproliferative, hepatoprotective, and analgesic effects [24]. Phytochemical analysis proved that alkaloids represent the major constituents of the genus, and some studies reported that they have anticancer effects. For Mahonia bealei and Mahonia oiwakensis, cytotoxic activity against human cancer cells was demonstrated [24, 25]. Previous studies also found in M. aquifolium extracts important quantities of alkaloids with cytotoxic effects on cancer cells [26]. One study demonstrated cytotoxic and antimetastatic effects of M. aquifolium stem-bark extract [27].

The phytochemical profile of plant extracts differs depending on the plant and the particular organ of a given plant [28]and of the extraction method [29]; the aim of the study was to test if M. aquifolium bark, leaf, flower, green fruit, and ripe fruit extracts can influence the TME in order to increase the antitumor responses. First, M. aquifolium extract immunomodulatory effects were tested on four human peripheral blood mononuclear cell (PBMC) subsets which have a key role in the adaptive immunity: CD4+ helper T cells, CD8+ effector T cells, CD19+ B cells, and CD14+ monocytes. Secondly, M. aquifolium extract antitumoral effects were tested on three cancer cell lines, DLD-1 colon carcinoma cells, A2780 ovary adenocarcinoma cells, and A375 malignant melanoma cell, and a nonimmune cell, BJ healthy skin fibroblast.

## 2. Materials and Methods

### 2.1. Plant Material

Fresh *Mahonia aquifolium* (Pursh) Nutt. bark, leaves, flowers, and fruits were purchased from the A. Borza Botanical Garden “Babes-Bolyai” University of Cluj-Napoca, Romania, between April and June 2018 and extracted in the Mycology Laboratory of “Babes-Bolyai” University, Cluj-Napoca, Romania, as previously described by a modified Squibb repercolation method with 70% ethanol (Merck, Bucuresti, Romania), producing the following extracts of *M. aquifolium*: bark extract 1 : 1.5 (g : mL) (1), leaf extract 1 : 1.2 (g : mL) (2), flower extract 1 : 1 (g : mL) (3), green fruit extract 1 : 1 (g : mL) (4), and ripe fruit extract 1 : 1 (g : mL) (5). The phytochemical analysis of the extracts has been performed and previously published [26, 30]. The plants were taxonomically identified and authenticated, and voucher specimens (number 665978) were deposited in “Alexandru Borza” Botanical Garden Herbarium, “Babes-Bolyai” University of Cluj-Napoca, Romania. The stock solutions were diluted with Phosphate-Buffered Saline Solution (PBS, from Sigma-Aldrich Company, St. Louis, USA), to obtain for each extract a series of stock concentrations from 500 to 10 *μ*g plant/mL.

### 2.2. Immunomodulatory Effects

#### 2.2.1. Isolation of PBMC Subsets

The biologic system used for testing was the suspensions of human PBMC, obtained by venipuncture from a 25-year-old healthy male volunteer, who gave his informed written consent before the blood collection, according to the approvals from the Ethical Committee of the Institute of Oncology “Prof. Dr. Ion Chiricuta” from Cluj-Napoca (IOCN), Romania, member of OECI. CD4+ helper T cells, CD8+ effector T cells, CD19+ B cells, and CD14+ monocytes were separated as previously described [31] (see supplemental data ([Supplementary-material supplementary-material-1])).

#### 2.2.2. PBMC Cytotoxicity Test

To assess cytotoxicity serial dilutions from *M. aquifolium* extracts, stock solutions were prepared, in order to obtain 5 successive concentrations, from 20 *μ*g/mL to 1 *μ*g/mL. The cytotoxicity of the extracts was assessed using the MTS viability dye (CellTiter 96 Proliferation Assay, manufactured by Promega Corporation, Madison, WI, USA), as previously described [31] (see supplemental data).

#### 2.2.3. Detection of IL-10-Positive and CD25-Positive Cells

After a prolonged exposure to the extracts 1-5 at a subcytotoxic concentration, CD4+, CD8+, CD19+, and CD14+ cell activation pathways through CD25 and interleukin-10 (IL-10), an immune-activating cytokine implicated in cancer immunotherapy [32], were evaluated by flow cytometry (see supplemental data).

#### 2.2.4. Tumor Necrosis Factor Alpha (TNF-*α*) Production

The soluble form of tumor necrosis factor alpha (TNF-*α*) production was measured, knowing that this inflammatory cytokine has an important role in tumor proliferation, metastasis, and neoangiogenesis [33]. The evaluation of the secreted TNF-*α* level through ELISA testing (kit acquired from Hycult Biotech, Uden, The Netherlands) was performed according to the manufacturer indication (see supplemental data).

### 2.3. Antitumoral Effect Evaluation

#### 2.3.1. Cell Cultures

The human cell lines used in the present study were DLD-1 colon carcinoma and the A2780 ovary adenocarcinoma cell lines acquired from the European Collection of Authenticated Cell Cultures (ECACC) through Sigma-Aldrich, St. Louis, USA; the A375 malignant melanoma and the BJ healthy skin fibroblast cell lines were from the American Type Culture Collection (ATCC) acquired through LGC Standards GmbH, Wesel, Germany (see supplemental data).

#### 2.3.2. Cytotoxicity Test

For the cytotoxicity testing, the colorimetric assay based on the tetrazolium dye 3-(4,5-dimethylthiazol-2-yl)-2,5-diphenyltetrazolium bromide (MTT) reduction to its purple colored formazan product was used. Because this process occurs only in the mitochondria of the living cells, the amount of living cells can be tracked by the color intensity of the samples. All experiments were performed in triplicates. The samples were analyzed as previously described [34] (see supplemental data).

#### 2.3.3. Protein Content of the Samples

For proteomic methods, the tumor and normal cells were seeded on 6-well plates at a concentration of 10^5^ cells/mL, and after 24 hours, they were treated with a 10 *μ*g/mL solution of each extract. The supernates and cell lysates were kept at -20°C until analysis. To evaluate the supernatants and cell homogenate total protein content, the Bradford assay was used. The calibration curve was prepared using seven serial dilutions starting from 100 *μ*g/mL proteins to 1.56 *μ*g/mL proteins (see supplemental data).

#### 2.3.4. Intracellular Caspase-3 and Caspase-8

The intracellular caspase-3 and caspase-8 were measured with an ELISA method, and the caspase concentration provided by the quantitative measurement (ng/mL) was normalized according to the total protein content of each lysate sample, and in this way, the caspase level was expressed as ng/mg protein, to ensure an accuracy of the assessment (see supplemental data).

#### 2.3.5. Soluble Intracellular Adhesion Molecule-1 (ICAM-1) and Vascular Cell Adhesion Molecule-1 (VCAM-1/CD106)

ICAM-1 was determined by using a human ELISA kit (E-EL-H2585 from Elabscience Biotechnology Co. Ltd., Houston, TX, USA), and VCAM-1/CD106 was measured with a human ELISA kit (E-EL-H5587, from Elabscience Biotechnology Co. Ltd., Houston, Texas) according to the manufacturer indications. ICAM-1 and VCAM-1/CD106 were expressed as ng/mg protein (see supplemental data).

#### 2.3.6. Matrix Metalloproteinase-9 (MMP-9)

MMP-9 was assessed with a human MMP-9 Platinum ELISA kit (MBS2016/2, from Affymetrix, through eBioscience, Vienna, Austria), according to the manufacturer's indications. MMP-9 was expressed as ng/mg protein (see supplemental data).

### 2.4. Statistical Analysis

GraphPad Prism 5 software (from GraphPad Software Inc., La Jolla, CA, USA) was used to compute IC50 values (nonlinear regression of the concentration versus normalized response) and Spearman nonparametric correlations, to compare values with the one-way analysis of variances, followed by Bonferroni or Dunnett multiple comparison posttest, and to compute the mean values and the standard error of the mean (SEM).

To examine the strengths of associations between the results, specifically Pearson correlations, we have used Statistica 12.0 for Windows (Stat-soft, Inc., USA). Multivariate data analysis was performed on the entire antioxidant and hematological parameters determined in this study using PCA (principal component analysis) incorporated in Statistica 12.0 software.

## 3. Results and Discussion

### 3.1. Immunomodulatory Effect

#### 3.1.1. PBMC Cytotoxicity Test

**Figure 1:**
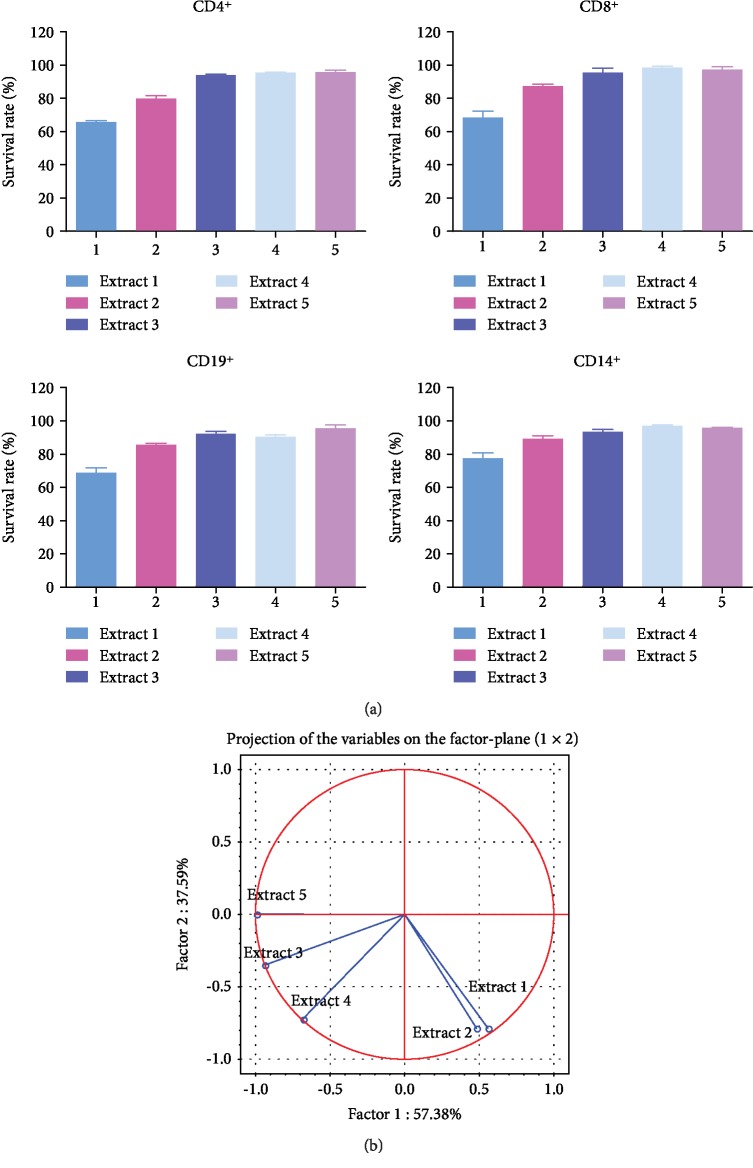
(a) Survival rate of PBMC subsets CD4+, CD8+, CD19+, and CD14+ treated for 24 hours with the *M. aquifolium* extracts 1-5, at a concentration of 10 *μ*g/mL in the cell culture media. (b) Correlation circle (loading plot) depicting the relationship between the extract outcomes on different PBMC subpopulations—a model obtained after applying principal component analysis.

From each *Mahonia aquifolium* extract, five concentrations were tested, between 1 and 20 *μ*g/mL in the cell culture media. All concentrations exhibited low cytotoxicity against PBMC, including the 20 *μ*g/mL concentration. The survival rates of the cells treated with the highest concentration were shown in Figure 1(a) (see supplemental data [Supplementary-material supplementary-material-1]).

In all PBMC subpopulations, *M. aquifolium* extracts 1-5 treatment induced a similar growth inhibition pattern. Extract 1 exerted the highest inhibitory effect, followed by 2 (Figure 1(b)), while 3, 4, and 5 caused less than 10% cell loss after 24-hour exposure. Consequently, for further testing, we used all extracts at the concentration of 20 *μ*g/mL. The principal component analysis (PCA) clearly indicated (Figure 1(b)) the similarities between 1 and 2 effects, these extracts being in the same quadrant of the loading plot, at distance to the group of extracts 3, 4, and 5, placed in another quadrant; further, 1 and 2 are distanced to more than 90 degrees to the least active 3 and 5 *M. aquifolium* extracts.

#### 3.1.2. IL-10-Positive and CD25-Positive Cells

The immunomodulatory effect of *M. aquifolium* extracts was evaluated by monitoring the number of CD25+ and IL10+ cells within a population of 10000 PBMC. CD25 and IL10 membrane marker expression was assessed by flow cytometry, and each data represents the median value of 10000 measurements, provided by the BD FACSDiva version 6.1 software. For each sample, two independent evaluations were made; their mean value was calculated with the column statistics, and the comparison between values was provided by the one-way analysis of variance in the 95% interval (Table 1) (see supplemental data Figures [Supplementary-material supplementary-material-1]).

Following the 24-hour exposure of the PBMC subsets to 1-5 *M. aquifolium* extracts, both the CD25 membrane marker and the IL-10 intracytoplasmic marker were modulated distinctly in each cell subpopulation. In untreated T cells, the basal level of the two molecules was modest, while in B lymphocytes and monocytes the basal values were higher (Table 1).

The effect of 1, the bark extract, was the prominent regulator of the lymphocyte activation through the CD25 pathway. Extract 1 distinguishes itself from other extracts through its lower chlorogenic acid content (see Supplemental data [Supplementary-material supplementary-material-1]) and through the presence of berbamine, jatrorrhizine, palmatine, and berberine, four compounds that are not present in the extracts 2 to 5 [26, 30].

Extract 1 upregulated the CD4+CD25+, CD8+CD25+, and CD14+CD25+ phenotype expression, and in this manner, 1 triggered the activation of both tumor suppressor and effector lymphocyte subsets and also the CD14+CD25+ monocytes, the main population of blood monocytes implicated in antitumor response [35]. CD14+ cells were proven to act against the tumor cells and the metastatic processes, directly by an antibody-mediated mechanism [36] and indirectly by activating the natural killer cells [37].

T cells which expressed CD4+ and CD25+ are essential in self-recognition and are known as T regulatory cells [38] and maintain the self-tolerance control in the immune response against infections, transplantation antigens, and tumor-associated antigens [39]. CD4+ T cells play antitumor roles through various mechanisms, and some studies claim that they could be efficient even in antitumoral immunotherapy like CD8+, previously known as the gold standard [40]. Although tumor-infiltrating CD4+CD25+ lymphocytes can contribute to the progression of the disease [41], they may act against tumors indirectly, helped by NK cells, or by inactivating the IL-10 pathway [42].

The fact that 1 exerts a significant enhancement of CD8+CD25+ activated phenotype converges to the antitumor potential of the bark extract, since CD8+ are important effectors of the antitumor cellular immunity in several cancers [43], counteracting the metastatic potential of tumor cells [44], and it was proven that their presence is related to the patient survival [45]. The increase of CD25+IL-10+ phenotype occurred following the treatments with 1, the only extract able to increase CD25, or concomitant with the increase of IL-10 induced by 2-5 in CD4+ and CD19+ (Table 1). IL-10 sustain the toxicity in CD8+ cells, [42] and in high concentrations, IL-10 is able to inhibit tumor growth by inhibiting the angiogenesis and the production of reactive oxygen species [46].

Extracts 2, 3, 4, and 5 could not influence significantly CD4+ or CD8+ cells. Instead, they influenced the double-positive populations and the IL-10 expression in these two subsets. Extract 2 increased the CD25+IL-10+ expression only on cytotoxic CD8+ T lymphocytes, without augmenting the helper CD4+ activation. Extracts 3 and 5 enhanced the CD25+IL-10+ activation in cytotoxic CD8+ cells but parallel in the regulatory CD4+ T cells. Overall, the PCA indicated that the outcome of the extracts on CD4+ subpopulation was opposed to CD8+, and the effect of 1-5 on CD14+ is opposed to CD19+ cells (Supplemental data [Supplementary-material supplementary-material-1]).

In CD19+ B cells, all extracts 1-5 performed likewise: they exerted a strong downregulatory effect on CD25+ cells, but the CD25+IL-10+ phenotype was upregulated (Table 1). The CD19+ expression is essential in the B cell-mediated immune response [47]. The increase of CD19+ cells and the regulatory CD19+CD25+IL-10+ B cell overexpression enhance the immunoglobulin production, but in tumors, it is responsible for metastatic growth support [48] as well, by costimulating the Treg cells. The CD19+CD25+IL-10+ phenotype was confirmed to be protector against inflammation [49] and promotes allograft survival in transplantation [50].

In CD14+, monocytes 2-5 had a strong downregulatory effect through CD25 and IL-10 decrease (Table 1), acting contrary to extract 1.

**Figure 2:**
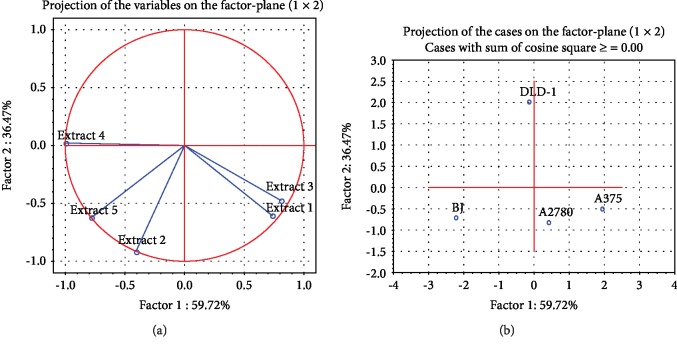
Principal component analysis on the extract half inhibitory concentrations: (a) correlation circle (loading plot) using the first two principal components of the PCA model obtained after applying tumoral cells (IC50) and (b) score plot using the first three principal components of 1-5 (tumoral cells) based on IC50.

The IL-10 expression decreased following the treatment with extract 1 in CD4+, CD8+ T cells, and monocytes and strongly upregulated in CD19+B cells (Figure 2). In IL-10 modulation as well, extracts 2, 3, 4, and 5 act convergently: they caused a significant increase of IL-10 phenotype in helper CD4+ T cells and CB19+ B cells and decline in effector CD8+ T cells and CD14+ monocytes.

It was a strong correlation between the CD25+ and CD25+IL-10+ phenotype in all subsets (Spearman nonparametric correlation, *p* < 0.0001) which means that the two subpopulations tend to increase or to decrease in the same time. In all PBMC subsets, the treatment with 1-5 induced no significant correlation between CD25+ and IL-10+ expression (nonparametric two-tailed correlation, Spearman *p* > 0.05).

#### 3.1.3. Tumor Necrosis Factor Alpha (TNF-*α*) Production

**Figure 3:**
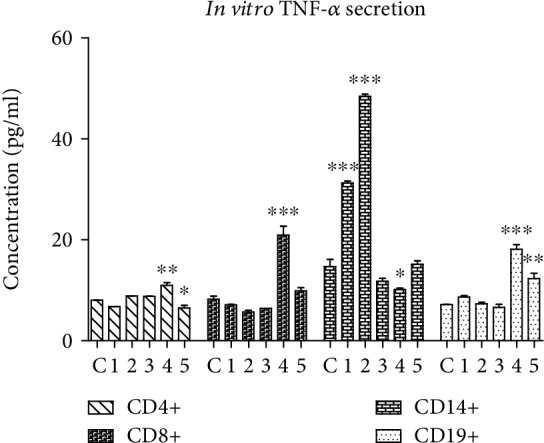
The *in vitro* effect of *M. aquifolium* extracts on soluble TNF-*α* secreted by the CD4+, CD8+, CD14+, and CD19+ cells after a 24-hour exposure to subcytotoxic doses. ^∗∗∗^Extremely significant differences between treated cells *vs*. untreated control, *p* < 0.001; ^∗∗^very significant differences between treated cells *vs*. untreated control, 0.001 < *p* < 0.01; ^∗^significant differences between treated cells *vs*. untreated control, 0.01 < *p* < 0.05.

TNF is a multifunctional cytokine that has important roles in cell survival, proliferation, differentiation, and death. As a proinflammatory cytokine, TNF may be implicated in inflammation-induced carcinogenesis. TNF exerts its functions by activating distinct nuclear factor-*κ*B (NF-*κ*B), an antiapoptotic signal, and c-Jun N-terminal kinase (JNK), a cell death signal. So, TNF is a double-edged sword with pro- or antitumorigenic effects [51], which in the early stages can contribute to antitumor response enhancement, while in late stages, it could maintain the tumor growth [52]. All PBMC subsets have had a certain basal TNF-*α* level (Figure 3), since CD4+ and CD8+ T lymphocyte subsets, B cells, and CD14+ monocytes are TNF-*α* secretory cells [53]. Following the treatment, TNF-*α* was modulated distinctively in each PBMC population. Extracts 1 and 2 had no effect on T or B cells, but they upregulate the TNF-*α* in CD14+ cells. In CD14+ monocytes, the main population of blood monocytes implicated in antitumor response, the direct cytotoxicity against the target cells is mediated by TNF-*α*, IL-12, reactive oxygen species, and reactive nitrogen species [35]. Since 1 and 2 modulated significantly the cytokine production only in CD14+ monocytes, it is expected that these two extracts will enhance the CD14+ cell antitumor capacity. Extract 3 was completely inert in the PBMC biologic system. Surprisingly, extract 4 obtained from green fruits significantly decreases the TNF-*α* production of monocytes, while in all the other studied subpopulations causes a very significant increase of TNF-*α*. Novel studies suggested that the increase of TNF-*α* secreted by the tumor-infiltrating T cells and monocytes may enhance the tumor cell death without associated systemic toxicities [52]. The TNF-*α* secreted by T lymphocytes can synergize with chemotherapy to strengthen tumor cell death mechanisms and the induced oxidative stress [54], and by enhancing the TNF-*α* production in CD4+ and CD8+ lymphocytes, extract 4 could balance the regulatory mechanisms induced through CD25. Extract 5 inhibited the TNF-*α* secretion in CD4+ T cells, and in B cells, a stimulation was observed.

Previous studies reported that in CD4+ and CD8+ T cells the IL-10+ phenotype is suppressed by exogenous TNF-*α* [55]. This was confirmed by the decrease of IL-10 positivity in CD8+ cells treated with 4, concomitant with the highest TNF-*α* secretion. It is known that TNF-*α* downregulates the function of tumor suppressor CD4+CD25+ lymphocytes [56], but overall, no correlation was found between TNF-*α* secretion and the IL-10+ or CD25+IL10+ expression.

As a conclusion, *M. aquifolium* extracts 1-5 exhibit moderate toxicity against PBMC subpopulations without selectivity towards any of the four studied subsets, and their modulator effect through CD25, IL-10, and TNF-*α* was well-balanced. The bark extract (1) effect was distinct, as regards the cytotoxicity, and its capacity to prime the helper CD4+ cells via CD25 activation, but this was compensated by the enhancement of CD8+ cytotoxic T lymphocyte activity. Also, 1 stimulated the immunoglobulin (the B cell secretor) through CD25 and IL-10 and triggered and amplified the TNF-*α* production in CD14+ monocytes only. The leaf extract (2), the richest in chlorogenic acid, rutin and isoquercitrin, has the capacity to activate the effector CD8+ lymphocytes and the B cells, deactivate the CD14+ monocytes, and in the same time increase a fold higher their TNF-*α* production.

**Figure 4:**
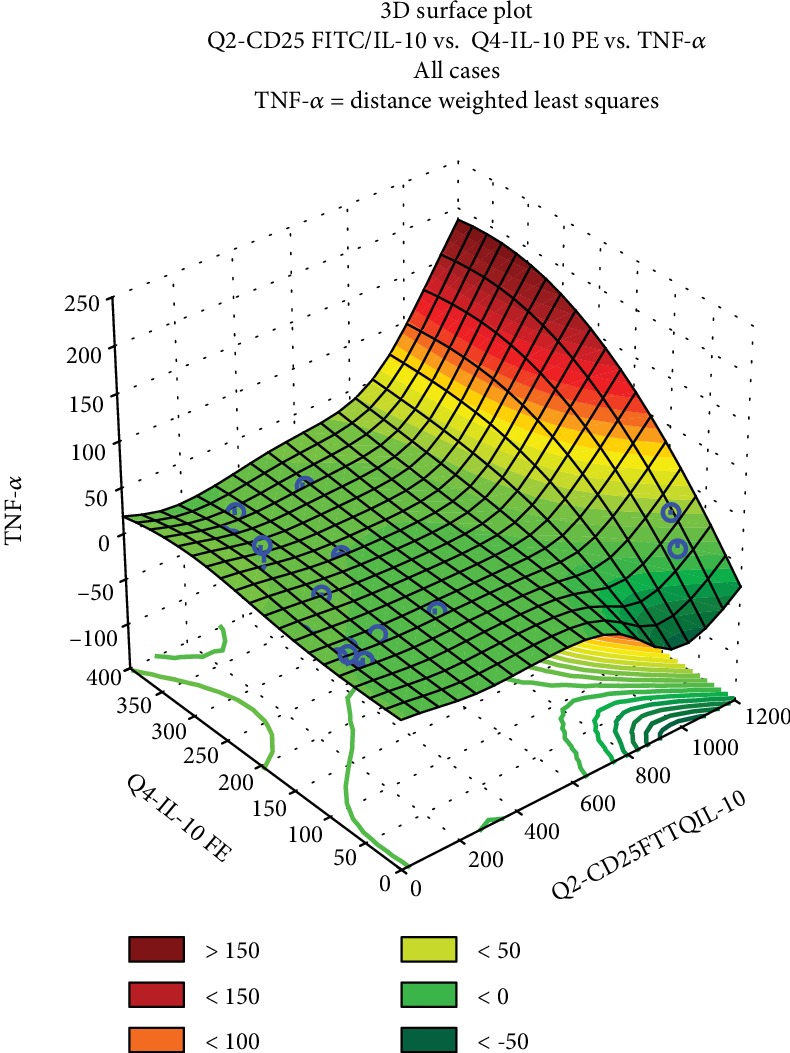
Dependence between the modulation of TNF-*α*, CD25, and IL-10 modulation for the studied Mahonia extracts, expressed as a tridimensional surface plot generated by the Statistica 12 principal component analysis (PCA).

TNF-*α* secretion was correlated with the CD25+ and CD25+IL-10+ phenotype but not with the CD25-IL-10+ cells (Supplemental data [Supplementary-material supplementary-material-1]). The PCA statistic confirms once again that CD25+ and CD25+IL-10+ populations were strongly correlated. In the tridimensional PCA (Figure 4), TNF-*α* variations were considered as a principal component; the concentrations of TNF-*α* were plotted with different colors, against the CD25 and IL-10 parameters. We can conclude that above the 100% increase of TNF-*α*, both IL-10 and CD25/IL-10 phenotype are overexpressed. Where the TNF-*α* is stationary towards the control values, then IL-10 is negatively correlated with CD25+ expression.

The flower extract (3) has the most moderate effect, it does not influence the T cells or monocyte activation or the TNF-*α* production; the only enhancement of activation was in B cells through the IL-10; therefore, the application of these extracts as part of a potential cancer treatment should not exhibit any unwanted effect on antitumor immunity.

There was a significant difference between green fruit extract 4 and ripe fruit extract 5, even if their composition is quite similar [26], the only notable difference being the higher chlorogenic acid level in 5. Extract 4 activates via IL-10 and not via CD25 the helper lymphocytes, deactivating the effector lymphocytes and monocytes. The only cell population which could be activated through 4 was the B subset, where the TNF-*α* production was also very high, costimulating the B cell responsiveness. Following the treatment with extract 5, the same outcome occurred at a smaller scale.

### 3.2. Antitumoral Effect

#### 3.2.1. Cytotoxicity Test

The toxicity of the *M. aquifolium* bark (1), leaf (2), flower (3), green fruit (4), and ripe fruit (5) extracts was assessed and quantified using the mathematic parameter half inhibitory concentration IC50, which represents the amount of compound necessary to eliminate 50% from the cells subjected to the treatment. Therefore, a small IC50 value is a characteristic for the toxic compounds (Table 2).

*In vitro*, extract 1 exhibited the best cytotoxicity, corresponding to the smallest IC50 value in the whole series (Table 2). This tendency was convergent in all cell lines, the IC50 values being around 10 *μ*g/mL, which is a moderate cytotoxicity according to the pharmacologic standards. Only extract 1 displays a dose-response relationship which can be described by the sigmoidal curve; in all the rest of extracts, the sigmoidal curves are distorted, because none of the tested concentrations was able to inhibit 100% of the cells (see supplemental data [Supplementary-material supplementary-material-1]). This effect can be correlated to the higher alkaloid content in the *M. aquifolium* bark extract [26]. Even the highest concentration of the extract was not able to inhibit 50% of the cells when the treatment was made with extracts 3 (in all cell lines), 4, and 5 (each in two cell lines); therefore, the IC50 values above 50 *μ*g/mL provided by the biostatistics software are hypothetical values, obtained by extrapolation.

The PCA statistics indicated few convergences in the extract IC50 values, when all the cell lines were analyzed (Figure 2(a)); A2780 and A375 outcome was well correlated, since the two values are in the same quadrant. The best selectivity of the extracts was observed in the DLD-1 and BJ contrast (Figure 2(b)).

Extract 2 has had the second-best cytotoxicity, but the IC50 values were twice as big as in 1. Extract 3 presented the lowest toxicity, while the IC50 values of 4 and 5 were in-between. The extract 2, but especially 4, showed a good selectivity towards the tumor cell lines, since in the normal BJ skin cell line, its IC50 value was higher than in tumor cells, which anticipate a lower toxicity towards normal skin fibroblasts following the administration versus the tumor cells with lower IC50. The cytotoxicity was dependent on the extracts and not on the cell type.

Based on the IC50 values (Table 2), for further testing, the 10 *μ*g/mL concentrations were used for each extract, ensuring in this way a good proportion of living cells for the mechanistic studies, even in the extract 1-treated cells.

#### 3.2.2. The Protein Content of the Samples

In order to identity the expression or production of certain molecules implicated in apoptosis, intercellular communication, and tumor dissemination, two types of samples were collected: the supernatants to identify soluble proteins secreted by the treated cells and cell homogenates, respectively, to identify the molecules implicated in the intracellular signaling. The protein content of the samples derived from supernatants and cell lysates was measured (Table 3) as described in Materials and Methods. The total protein concentration (*μ*g/mL) itself is not an indicator of cytotoxicity; instead, it was used to normalize the quantity of the target proteins after they were measured by immune-enzymatic methods (see supplemental data [Supplementary-material supplementary-material-1] and [Supplementary-material supplementary-material-1]).

#### 3.2.3. The Extrinsic Apoptosis Induction

The caspase cascade is the basic apoptotic signaling pathway, involved in both intrinsic and extrinsic apoptosis. Caspases are essential components of the apoptotic process. Caspase-8 is an initiator caspase of the extrinsic apoptotic pathway, while caspase-3 has an effector role, being activated by caspase-8 or other caspases or chemokines.

Some of the studied *M. aquifolium* extracts were capable to enhance the TNF-*α* expression in PBMC. On the extrinsic apoptotic pathway, the TNF-*α* increase implicates the proteolysis or cleavage of caspase-8, which further can activate caspase-3. Therefore, the intracellular level of these two caspases was evaluated following the 24-hour *in vitro* treatment of tumor (A2780, DLD-1, and A375) and normal (BJ) cell lines with extracts 1-5. The active or cleaved form of the two proteins is of interest in the apoptotic process, and the increase of the cleaved caspase expression is known to be an indicator of the apoptotic process (data provided by Metacore of Clarivate Analytics: Apoptosis Pathway Map, http://pathwaymaps.com/maps/373).

**Figure 5:**
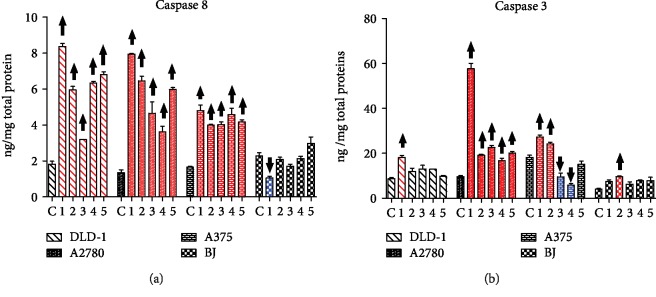
The intracellular level of the active initiator caspase-8 and the effector caspase-3 following the *in vitro* treatment with extracts 1-5. ^↑^Significant increase of caspase-8 or caspase-3 level; ^↓^significant decrease of caspase-8 or caspase-3 level compared with the untreated control.

The apoptotic process initiation through caspase-8 occurred in all tumor cell lines for all extracts 1-5 (Figure 5), with statistical significance in the 95% confidence interval (one-way analysis of variance, Dunnett posttest). In normal BJ cells, only extract 1 effect was significant, but in the opposite direction, meaning caspase-8 decrease. This indicates a selectivity of the extracts regarding the cell death mechanism induction.

The caspase cascade was evolving through caspase-8 towards the programmed cell death, but not in all treatments, or in all cell lines, the proapoptotic signal was propagated. The active caspase-3 increased following the exposure to 1 in all tumor cell lines, and 2 induced and increased in DLD-1 and A375 cells, and this denotes that the caspase cascade was influenced by 1 and 2 to evolve towards the extrinsic apoptotic pathway. In DLD-1 colon carcinoma, even 3, 4, and 5 caused caspase-3 cleavage; therefore, it is very likely the extrinsic apoptosis triggered by any of extracts 1-5 in K-ras mutant aggressive DLD-1 cells. In BJ cells, only extract 2 augmented the caspase-3 expression.

It was found that berberine, the main alkaloid from *M. aquifolium* extracts, mostly the bark extract, enhanced the expression and activation of caspase-3 and caspase-8 in ovarian cancer cells [57]. This finding may be an explanation for the highest caspase activation by extract 1 with the most important berberine content [30]. Once again, extract 1 was constantly active against all cell lines and selective towards normal BJ cells.

#### 3.2.4. Modulation of Adhesion Molecules

The intracellular adhesion molecule-1 (ICAM-1) and the vascular adhesion molecule-1 (VCAM-1) are implicated in tumor growth, extravasation, and dissemination. ICAM-1 is expressed on fibroblasts, keratinocytes, endothelial cells, and leukocytes, but also in many types of tumors [58]. ICAM-1 enhances tumor cell adherence to endothelial cells favoring tumorigenesis [59]. Moreover, it was reported an association between tumor relapse or drug resistance and ICAM-1 overexpression [60]. In inflammation, VCAM-1 is predominantly expressed on endothelial cells, but in cancer, it plays a dual role: increases the ability to metastasize and rises tumor-associated monocytes and macrophage recruitment. In tumors like breast, renal, and gastric carcinoma, aberrant VCAM-1 expression was found on tumor-associated vasculature and on tumor cells. In other tumor-associated vessels of some human malignancies has been reported downregulation of ICAM-1 and VCAM-1 [58]. The soluble form of the two proteins can act as biomarker in clinical diagnosis and treatment follow-up, and generally, the elevated values indicate a poor prognosis.

**Figure 6:**
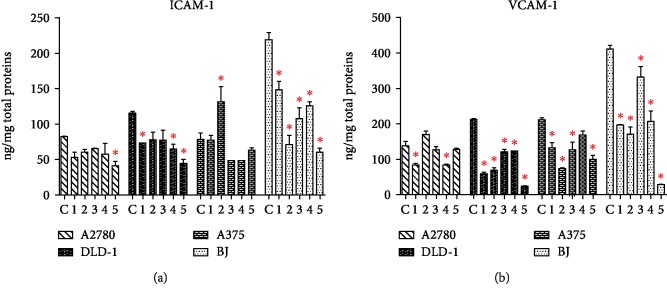
The modulation of adhesion molecules ICAM-1 and VCAM-1 implicated in the cancer cell extravasation and metastasis. ^∗^Significant decrease of ICAM-1 or VCAM-1 following the 24-hour treatment with 1-5, compared with the untreated control.

The extracts did not act identically against the ICAM-1 secreted by tumor cells (Figure 6). Extract 1 acts only on DLD-1 cells, generating a significant drop of ICAM-1; 3 influenced only the A375 cell line, while 4 and 5 were efficient in DLD-1 cells.

The extracts 1-5 were more active against VCAM-1, and in this case, extract 1 was capable to reduce the VCAM-1 secretion in all tumor cell lines (A2780, DLD-1, and A375). Extract 2 and even the less active extract 3 reduced VCAM-1 in DLD-1 and A375 cells.

Interestingly, all extracts have had an inhibitory effect against the ICAM-1 and VCAM-1 secreted by BJ normal fibroblasts *in vitro*. This property could be used to initiate further studies, since the elevated ICAM-1 or VCAM-1 levels are a characteristic of benign skin disorders; therefore, the *M. aquifolium* extracts gain a new perspective for future applications.

**Figure 7:**
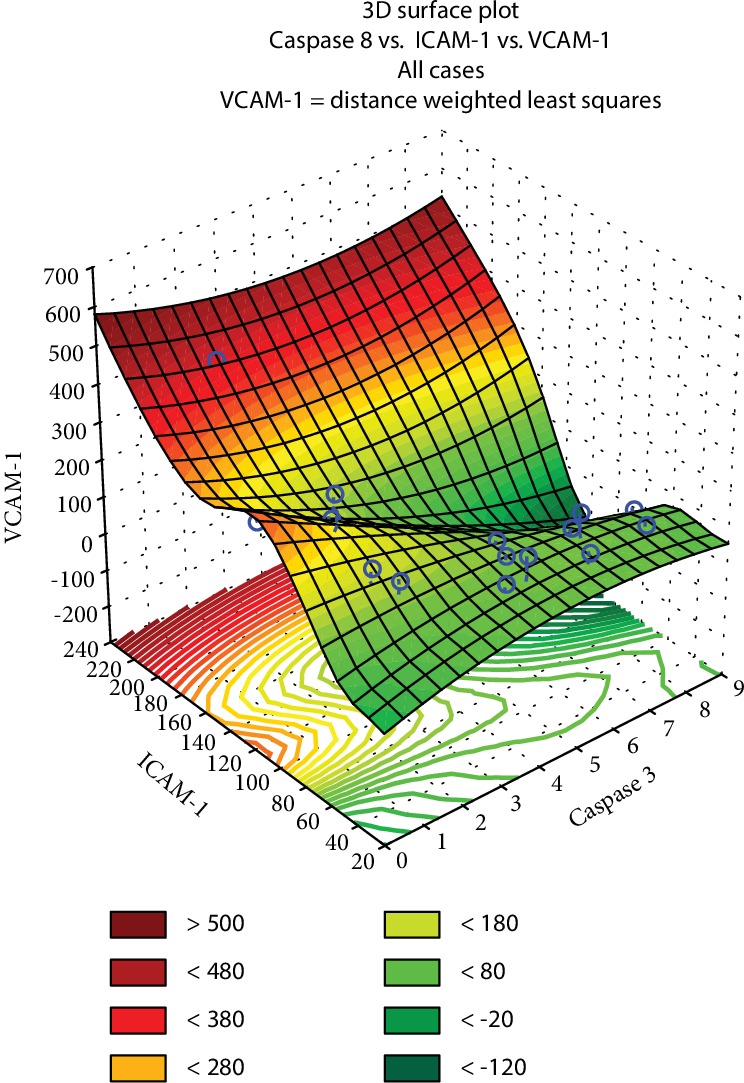
The relationship between the apoptotic effect and the influence on cell adhesion for the *Mahonia aquifolium* extracts 1 to 5, determined through principal component analysis for the extracts 1 to 5.

The PCA model is a useful tool to examine the multifaceted biologic effect of many natural extracts [61]. This method highlighted the relationship between the 1-5 extract capacity to trigger apoptosis and to influence the adhesion molecules which give the tumor cells the capacity to migrate (Figure 7): for strong VCAM-1 increase, up to 380 units, the expression of caspase-3 and ICAM-1 was directly proportional, while to a moderate increase of VCAM-1, the two parameters were divergent. VCAM-1 could increase up to 400 units, or it could be diminished up to 800-unit weight against the untreated controls in different tumor cell types, at the same time as the correlation remains indirect between ICAM-1 and caspase-3 (Supplemental data [Supplementary-material supplementary-material-1]).

#### 3.2.5. The Effect on Matrix Metalloprotease-9 (MMP-9)

**Figure 8:**
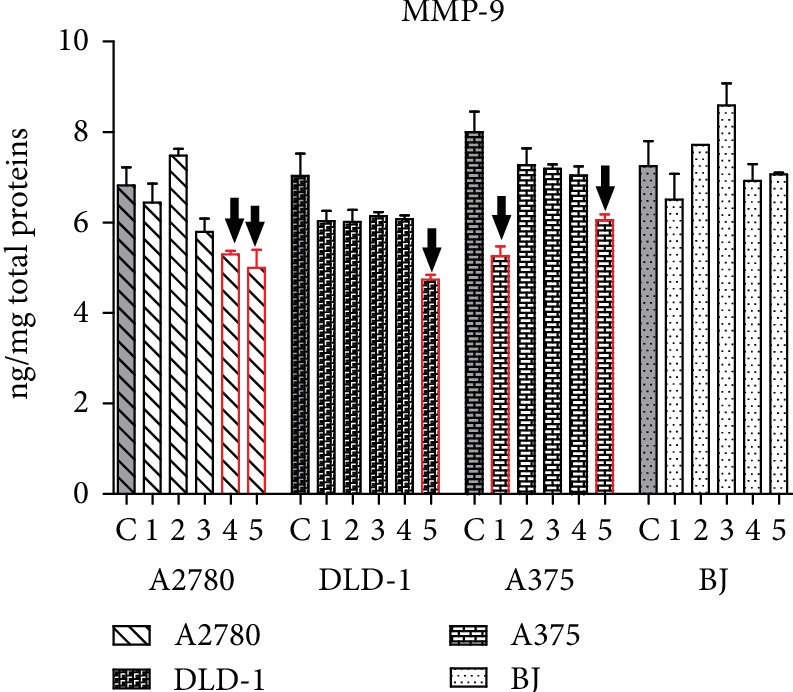
Modulation of MMP-9 by the treatment with extracts 1-5. ^↓^Significant decrease of MMP-9, compared with the untreated control.

MMPs, synthesized by neoplastic and stromal cells, are zinc-dependent proteases, which play a role in extracellular matrix remodeling and cancer progression, invasion, and metastasis. Because MMP-9 has been found overexpressed in tumor tissues compared with the adjacent nontumor tissues, it can be a prognostic molecular biomarker [62]. Against MMP-9 overexpression, the best cytotoxic extracts 1 and 2 do not act in a significant manner, and 3 as well has no effect (Figure 8). The only exception is 1, which was able to inhibit MMP-9 in melanoma cell A375. Instead, extracts 4 and 5 were more active against the MMP-9 overgrowth. Extract 5 inhibited MMP-9 in all tumor cell lines, while 4 was active only in A2780. In normal BJ cells, none of the treatments caused significant drop in MMP-9 levels.

**Figure 9:**
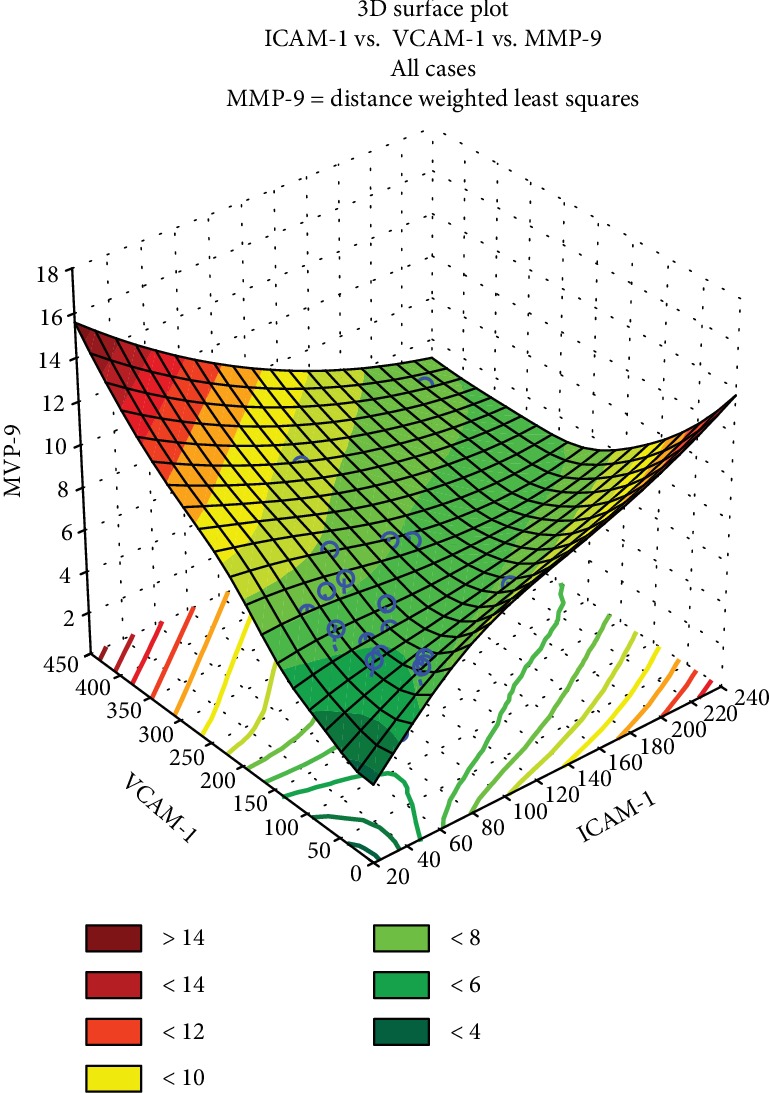
Tridimensional PCA model regarding the dependence between matrix metalloproteinase MMP-9 versus ICAM-1 and VCAM-1 adhesion molecules for the studied *Mahonia aquifolium* extracts 1-5.

Following to the treatment with 1-5 if MMP-9 values were amplified (above 12 units), then ICAM-1 and VCAM-1 were both elevated (Figure 9). Instead, if MMP-9 secretion was moderate, below 10 units, one of variables ICAM-1 and VCAM-1 has increased, simultaneously with the decline of the other parameters.

## 4. Conclusion

In conclusion, the extracts 1-5 have good potential to become TME modulatory agents, similar with other *Mahonia* extracts [27] described before, due to the immunomodulatory and antitumoral effects. Extract 1 should be employed carefully, and further tested, to estimate whether the increase of the regulatory helper CD4+cell level is counterbalanced with the tumor-suppressing mechanisms *in vivo*. The extracts 2, 4, and 5 are more suitable to activate the effector CD8+ cells, the monocytes, and B cells, and the utilization of 3 more likely will substantially benefit together with other active compounds, since it will give no secondary effects in the case of systemic administration, together with the standard antitumor drugs or alone. The *M. aquifolium* extracts also exhibited a moderate cytotoxicity on tumor cells. Among them, extract 1 was prominent as regards the antiproliferative capacity. We demonstrated for the first time that at subcytotoxic concentrations, the tumor cell lines subjected to 1-5 undergo changes in the signaling pathways linked to cell adhesion, proliferation, migration, and apoptosis (Table 4, Figure 9). These results open perspectives to further investigation of the *M. aquifolium* extract prodrug potential.

## Figures and Tables

**Table 1:** The *in vitro* effect of *M. aquifolium* extracts 1-5 on CD25 membrane marker and intracellular IL-10 expression in CD4+, CD8+, CD14+, and CD19+ cells.

Cell types	Q1—CD25 FITC		Q2—CD25 FITC/IL-10 PE		Q4—IL10 PE	
	Median values	SD	Median values	SD	Median values	SD
CD4+						
Untreated	23.5	0.71	62.5	2.12	86.5	2.12
Extract 1	301.0^∗∗∗^	12.73	1108.5^∗∗∗^	4.95	53.0^∗∗∗^	1.41
Extract 2	12.0	1.41	57.5	3.54	113.5^∗^	7.78
Extract 3	12.0	2.83	180.5^∗∗∗^	0.71	333.0^∗∗∗^	2.83
Extract 4	17.5	3.54	154.5^∗∗∗^	6.36	243.0^∗∗∗^	12.73
Extract 5	7.5	0.71	136.5^∗∗∗^	2.12	158.5^∗∗∗^	2.12
CD8+						
Untreated	3.0	0.00	58.0	2.89	162.5	9.19
Extract 1	119.5^∗∗∗^	0.71	396.0^∗∗∗^	1.41	113.0^∗∗∗^	0.00
Extract 2	2.0	0.00	114.5^∗∗∗^	6.36	122.0^∗∗∗^	0.00
Extract 3	1.5	0.71	61.5	0.71	148.0	4.24
Extract 4	1.0	0.00	55.5	6.36	95.5^∗∗∗^	0.71
Extract 5	2.5	0.71	97.0^∗∗∗^	0.00	91.5^∗∗∗^	0.71
CD14+						
Untreated	51.0	1.41	268.0	2.83	349.0	1.41
Extract 1	310.5^∗∗∗^	0.71	1131.0^∗∗∗^	1.41	71.5^∗∗∗^	2.12
Extract 2	9.5^∗∗∗^	0.61	129.5^∗∗∗^	0.71	248.0^∗∗∗^	2.83
Extract 3	12.5^∗∗∗^	2.12	188.0^∗∗∗^	2.83	320.0^∗∗^	12.73
Extract 4	22.5^∗∗∗^	3.55	167.5^∗∗∗^	3.54	179.5^∗∗∗^	2.12
Extract 5	8.5^∗∗∗^	2.12	189.5^∗∗∗^	2.12	188.0^∗∗∗^	2.83
CD19+						
Untreated	90.0	0.00	209.0	1.41	118.5^∗∗∗^	9.19
Extract 1	81.0	1.41	582.5^∗∗∗^	3.54	241.5^∗∗∗^	7.78
Extract 2	11.5^∗∗∗^	2.12	322.0^∗∗∗^	2.83	290.5^∗∗∗^	0.71
Extract 3	33.0^∗∗∗^	2.83	358.0^∗∗∗^	5.66	230.0^∗∗∗^	0.00
Extract 4	29.0^∗∗∗^	1.41	481.5^∗∗∗^	9.19	338.5^∗∗∗^	4.95
Extract 5	21.0^∗∗∗^	1.41	359.5^∗∗∗^	0.71	232.5^∗∗∗^	3.54

^∗∗∗^Extremely significant differences between treated cells *vs*. untreated control, *p* < 0.001. ^∗∗^Very significant differences between treated cells *vs*. untreated control, 0.001 < *p* < 0.01. ^∗^Significant differences between treated cells *vs*. untreated control, 0.01 < *p* < 0.05.

**Table 2:** Half inhibitory concentrations (IC50) of *M. aquifolium* extracts 1-5 on tumor and normal cell lines after 24-hour *in vitro* treatment, expressed as median value (*μ*g/mL) ± standard error (SD) of logIC50. The median values were extracted from the dose-response sigmoid curve (logarithm of concentration versus growth inhibition percent, analysis performed with the GraphPad Prism 5 software) (see supplemental data [Supplementary-material supplementary-material-1]).

	Extract 1		Extract 2		Extract 3		Extract 4		Extract 5	
	Median values	SD	Median values	SD	Median values	SD	Median values	SD	Median values	SD
DLD-1	9.67	0.05	14.26	0.07	56.05^∗^	0.11	67.01^∗^	0.08	32.63	0.10
A2780	12.72	0.06	22.59	0.08	72.23^∗^	0.11	57.16^∗^	0.09	52.59^∗^	0.12
A375	15.76	0.05	19.85	0.06	70.89^∗^	0.05	26.86	0.08	36.42	0.06
BJ	11.03	0.04	25.06	0.05	53.55^∗^	0.15	102.81^∗^	0.22	73.01^∗^	0.11

The values above 50 *μ*g/mL are hypothetical; they were obtained by extrapolation, given that the highest concentration used in the experiment was 50 *μ*g/mL.

**Table 3:** The protein content (*μ*g/mL) of the supernatants and cell lysates harvested from the cell cultures after the 24-hour treatment with 1-5.

	Supernatant		Cell lysate	
	Avg.	SD	Avg.	SD
DLD-1 untreated	178.17	10.17	276.84	18.41
DLD-1 treated with extract 1	209.33	15.61	291.17	20.22
DLD-1 treated with extract 2	173.11	11.81	302.94	22.17
DLD-1 treated with extract 3	182.00	12.50	285.00	27.75
DLD-1 treated with extract 4	196.94	9.35	279.06	7.10
DLD-1 treated with extract 5	230.50	9.09	279.83	3.44
A2780 untreated	157.00	25.07	299.06	7.70
A2780 treated with extract 1	169.89	15.79	313.06	29.48
A2780 treated with extract 2	188.03	37.70	301.17	26.07
A2780 treated with extract 4	210.61	10.30	260.09	25.58
A2780 treated with extract 5	215.56	12.86	292.17	26.10
A375 untreated	261.61	36.76	269.75	41.13
A375 treated with extract 1	198.06	16.39	275.67	44.74
A375 treated with extract 2	203.94	17.70	278.67	36.06
A375 treated with extract 3	236.67	4.82	300.17	52.50
A375 treated with extract 4	212.11	15.89	275.34	20.98
A375 treated with extract 5	192.44	38.60	279.89	32.49
BJ untreated	199.50	21.36	274.39	26.58
BJ treated with extract 1	207.45	1.11	281.09	0.83
BJ treated with extract 2	197.78	31.59	283.34	12.96
BJ treated with extract 3	174.67	14.78	296.44	15.59
BJ treated with extract 4	214.17	5.20	268.06	32.07
BJ treated with extract 5	212.61	17.74	191.61	14.43

**Table 4:** Significant modulations of the antitumor signaling following the *in vitro* treatment of tumor cell populations with *M. aquifolium* extracts 1-5.

*Cell line*	*A2780*					*DLD*					*A375*				
Extract	1	2	3	4	5	1	2	3	4	5	1	2	3	4	5
Caspase-8	+	+	+	+	+	+	+	+	+	+	+	+	+	+	+
Caspase-3	+					+	+	+	+	+	+	+			
ICAM-1					+	+			+	+		+			
VCAM-1	+			+		+	+	+	+	+	+	+			+
MMP-9				+	+					+	+				+

## Data Availability

Data are available in the manuscript and supplemental data file.

## Supplementary Materials

Supplementary Materials More information about the methods, materials, and results can be found in the Supplemental data folder, as indicated in the main text.
