# Supplementary material for: Antitumoral and Immunomodulatory Effect of Mahonia aquifolium Extracts
Source: Oxid Med Cell Longev. 2019 Dec 14;2019:6439021. doi: 10.1155/2019/6439021 (PMC6948282; doi:10.1155/2019/6439021)
Supplement: Supplementary Materials — More information about the methods, materials, and results can be found in the Supplemental data folder, as indicated in the main text. [file 6439021.f1.docx]

**Supplemental data**

**Antitumoral and Immunomodulatory effect of *Mahonia aquifolium* extracts**

Andra Diana Andreicuț^1^, Eva Fischer-Fodor^2,3^, Alina Elena Pârvu^1^, Adrian Bogdan Ţigu^2,5^, Mihai Cenariu^4^, Marcel Pârvu^5^, Florinela Adriana Cătoi^1^, Alexandru Irimie^6^

^1^Pathophysiology, Faculty of Medicine, University of Medicine and Pharmacy Iuliu Hațieganu, RO-400012, Cluj-Napoca, Romania

^2^Medfuture Research Center for Advanced Medicine, University of Medicine and Pharmacy Iuliu Hațieganu, RO-400012, Cluj-Napoca, Romania

^3^Tumor Biology Department, The Oncology Institute I. Chiricuță, RO- 400015, Cluj-Napoca, Romania

^4^ Biotechnology Research Center, University of Agricultural Science and Veterinary Medicine, Cluj-Napoca, Romania

^5^Faculty of Biology and Geology, Babeș-Bolyai University, RO- 400015, Cluj-Napoca, Romania

^6^Oncology, Faculty of Medicine, University of Medicine and Pharmacy Iuliu Hațieganu, RO-400012, Cluj-Napoca, Romania

Correspondence should be addressed to Eva Fischer-Fodor; [fischer.eva@iocn.ro](mailto:efischerfodor@yahoo.com). Alina Elena Pârvu; [parvualinaelena@umfcluj.ro](mailto:parvualinaelena@umfcluj.ro)

**Material Immunomodulatory effect**

*Isolation of PBMC cell subsets*

The blood was collected in Lithium-Heparin Vacutainer blood collection tubes (from Beckton, Dickinson and Company Europe, Heidelberg, Germany). PBMC were isolated as described before, by gradient density method: the whole blood was mixed 1:1 with Phosphate Buffered Saline Solution (PBS), slowly transferred onto a Histopaque 1.077 layer and centrifuged at 2000 RPM, 15 minutes in a swing-out rotor of the Hettich 320 centrifuge (from Hettich Lab Technology, Tuttlingen, Germany). The lymphocytes were washed in Hank’s Buffered Salt Solution (HBSS), then resuspended in a buffer which consists of 0.5% fetal calf serum (FCS) in PBS. Histopaque, HBSS, PBS and FCS were acquired from Sigma Aldrich Company, St Louis, USA.

The cells were subjected to magnetic separation using a MACS Multistand, MiniMACS separators and CD4, CD8, CD14 and CD19 microbead kits (all materials and kits provided by MiltenyiBiotec, BergischGladbach, Germany), according to the manufacturer’s instructions. For each separation, 10^7^ PBMC’s were suspended in 80µL separation and incubated with 20 µL antibody-labeled magnetic microbeads for 15 minutes at 2-8 Celsius degrees. After washing in cold separation buffer, cells were resuspended in 500 µL buffer and the positively marked population was separated on MS columns (from MiltenyiBiotec).

The CD4+, CD8+, CD19+ and CD14+ populations resulted from the separation processes were washed with HBSS, the cells were counted with the Automated Cell Count Eve (from NanoEnTek, Seoul, Korea) and resuspended in RPMI-1640 cell culture media supplemented with 10% FCS, 1% non-essential aminoacids solution, 1x sodium piruvate solution, 1mM glutamine, Hepes buffer and mercaptoethanol solution (all media and supplements were from Sigma Aldrich Company).

*Cytotoxicity test*

The suspensions of PBMC subsets in above described RPMI-1640 based media were dispensed into clear bottom 96-well microplates (from Nalgene Nunc, through Thermo Scientific Company, Waltham, MA, USA). 100μL of suspension was placed into the well, containing 5x10^4^ CD4^+^, CD8^+^, CD19^+^ or CD14^+^ cells; only one cell type was seeded to each plate. After 24 hours of incubation at 37°C in a humidified incubator with 5% CO_2_ atmosphere (Steri-Cycle i160 CO_2_ incubator from Thermo Scientific, Cleveland, USA), each well was treated with 5 μL of *M. acquifolium* extracts **1**-**5**. From each extract 5 concentrations were tested in triplicates. The control wells were treated with 5 μL PBS. After 24 hours exposure to the extracts **1**-**5**, to each well 10 μL of MTS-PMS mix was added, according to the manufacturer’s indications, and after 4 hours of incubation, colorimetric measurements were performed with the Synergy2 microplate reader (from BioTek Company, Winooski, VT, USA) at 570nm. As references, untreated cells were used, and as blank, cell culture media; the blank values were substracted from the absorbance values obtained and for each concentration, the survival rate was calculated. Using the GraphPad Prism5 biostatistic software (acquired from GraphPad Software, La Jolla, CA, USA), the survival rates were compared with the untreated cells to which 100% survival was attributed, and a linear regression curve was drawn for each extract.

*Detection of IL-10 positive and CD25 positive cells*

The CD4+, CD8+, CD19+ and CD14+ cell populations were seeded on 12-well Nunclon plates (Nalgene Nunc, through Thermo Scientific Company, Waltham, MA, USA), in a concentration of 3x10^5^ cells/mL media. The cells were incubated for 24 hours at 37 °C and 5% CO_2_ in a humidified Steri-Cycle i160 CO_2_ incubator (from Thermo Scientific, Cleveland, USA). For each cell population 12 wells were prepared with 1470 µL suspension of CD4+, CD8+, CD14+ or CD19+ cells. The cells were treated with *M. acquifolium* extracts **1**, **2**, **3**, **4** and **5;** 30µL extract was added into each well, to obtain a final concentration of 20µg/ml of extract in the cell’s suspension. From all treatments, duplicates were used (two individual wells for each treatment). As reference we used untreated cells collected from two distinct wells.

After 24-hours treatment, the cells were harvested from the plates by pipetting. The adherent CD14+ cells were removed gently, using a cell scrapper. The cells were centrifuged (using the 32R centrifuge, Hettich Lab Technology, Tuttlingen, Germany) then washed with cold wash buffer (BD Cell Wash solution from Becton Dickinson Biosciences, Franklin Lakes, NJ, USA) by centrifugation at 1200 RPM, for 10 minutes at 37 °C degrees. Cells were resuspended in 90 µL cold RPMI medium, 10 µl of IL-10 Catch reagent was added (component of IL-10 Cell Enrichment and Detection kit, from Miltenyi Biotec, Bergisch Gladbach, Germany), and the cells were incubated for 5 minutes on ice. The cells were diluted with 1ml warm medium (37 °C) and incubated for 45 minutes under slow continuous rotation on the platform of the orbital shaker-incubator ES-20 (from Biosan, Riga, Latvia). After washing with cold buffer, the cells were resuspended in 90 µl cold buffer and labeled with phycoerythrin (PE)-conjugated IL-10 detection antibody (component of the IL-10 Cell Enrichment and Detection kit). Additionally, FITC anti-CD25 antibody (from Becton Dickinson & Company, Franklin Lakes, NJ, USA) was added, 25 µl in each tube. After 10 minutes of incubation on ice, the cells were washed and resuspended in 500 µl cold buffer, then analyzed by flow-cytometry using the FACS Canto II equipment (from Becton Dickinson & Company; Franklin Lakes, NJ, USA).

*Tumor necrosis factor alfa (TNF-α) production*

The cells were treated as described above, collected from the plates and centrifuged in a cooled centrifuge at 1200 RPM for 10 minutes. The supernates were harvested, centrifuged at 2000 RPM for 10 minutes and stored at -80 Celsius degrees in cryovials (from Nunclon). The samples were brought to room temperature before the testing, together with the components of the kit. From each sample, aliquots of 100µL were transferred to antibody-coated microwells on the testing plate; duplicates were processed. Serial dilutions were prepared from the standard human TNF-α lyophilized protein provided by the manufacturer, and 100µL from each solution was dispensed on the testing plate. The dilution buffer from the kit was used as blank. The plate was incubated for one hour at room temperature, then washed four times with washing solution provided by the kit, using an automated microplate washer (from DiaSource Immunoassays, Louvain-La-Neuve, Belgium). 100µL of secondary, or tracer antibody was added to the wells, the assay plate was incubated for one hour, and then washed four times. Into each well 100µL of streptavidin-peroxidase solution was pipetted, incubated, then washed four times, and finally 100µL of TMB substrate was added. The plate was incubated for 30 minutes at room temperature, stop solution was added and the assay plate was read at 450nm, with reference at 540nm, using the Sunrise microplate reader with Magellan software (equipment and software from Tecan Group, Männedorf, Switzerland). The software built the standard curve and provided the individual absorbance data and the individual concentrations of TNF-α for each sample.

***Antitumoral effect evalua******tion***

*Cell cultures*

The DLD-1 and A2780 cells were cultivated in RPMI-1640 cell culture media, the BJ cells in Eagle’s Minimal Essential Medium (MEM), all media supplemented with 10% fetal bovine serum (FCS). The A375 malignant melanoma cell line was cultivated in Dulbecco's Modified Eagle's medium (DMEM) with 4500 mg/ml glucose, supplemented with 10% FCS (all media and supplements were from Sigma Aldrich Company, St. Louis, USA).

The *in vitro* experiments were performed in a fully equipped cell culture laboratory, having the basic equipment: Thermo Scientific Steri-Cycle i160 CO2 Incubators (from Thermo Scientific, Cleveland, USA); MPW-352-R centrifuge with swing-out rotor (from MPW Med Instruments, Warsaw, Poland), Esco Class II BSC laminary hoods (acquired from Esco, Changi, Singapore); Synergy2 plate reader (from BioTek Company, Winooski, USA), Zeiss Observer D1 inverted phase fluorescence microscope (Oberkochen, Germany); Tecan Sunrise Elisa reader (Tecan Group, Grödig/Salzburg, Austria); Plus centrifuge DLAB (from Dragon Laboratory Instruments, Beijing, China); orbital shaker-incubator ES-20 (from Biosan, Riga, Latvia); Biobase MW9623 Micro plate washer (from Biobase, Shandong, China) and ULF ultrafreezer 480W PRO2 (from EVERmed, Motteggiana, Italy).

*Cytotoxicity test*

The A2780, DLD-1, A375 and BJ cells were seeded on 96-well flat bottom microplates at a concentration of 2x10^4^ cells/well in 190μL suitable cell culture media. After 24 hours, when the cells were attached to the cell culture plate surface and the proliferation begun, the cells were treated by adding 10 μL of *M. aquifolium* extract onto the cells in each well. For each extract seven different concentrations were used, and for reference cells 10 μL of PBS. Since the cell culture media : extract solution ratio was 20:1, the final concentrations of the extracts were between 50 and 0.1μg/mL.The cell culture media was discarded from the wells of the microplate, then 100 µL of 1mg/mL MTT (from Merck KGaA through Sigma-Aldrich, St. Louis, USA) solution was added to each well, and incubated at 37ºC. After one hour of incubation, the MTT solution was discarded, and replaced with 150 µL of dimethyl sulfoxide (from Titolchimica, Pontecchio Polesine, Italy) to dissolve the formazan crystals formed inside the cells. The intensity of coloration was measured immediately with the BioTek Synergy2 multiplate reader at 570nm wavelength. The absorbance values were employed to determine the half inhibitory concentration (IC50) values from dose-response curves generated by the GraphPad Prism 5. IC50 was used to establish the subcytotoxic concentration to be used for the study of the cell death mechanisms and the signaling pathways.

*Protein content of the samples*

The supernatants were collected, centrifuged at 2000 RPM for 10 minutes, aliquot and kept in freezer at -20 ºC. After the supernatants were removed, the plates were washed twice with PBS, the cells were harvested by trypsinization, washed in PBS and to each sample 1 mL of cell lysis solution was added (NP40 cell lysis buffer, from Invitrogen, through Thermo Fischer Scientific, Waltham, MA, USA). The samples were kept on ice for 30 minutes, they were kept in sonicator bath for 5 minutes, then centrifuged, and aliquot similar with the supernatants.

For the Bradford assay a series of samples including supernatants and cell homogenates of the treated A2780, A375, DLD-1 and BJ cells were prepared in 1.5 mL eppendorf tubes, in triplicates, using 100 μL of each sample with 400 μL of Bradford reagent, prepared in-house based on the acidified Coomassie Blue (from Sigma Aldrich) solution, having red color, which turns to its anionic blue form after the reaction with the amino acids from the proteins. After adding the Bradford reagent, 200 μL of the samples were transferred in a transparent 96 well flat-bottomed plate and analyzed using a Tecan Spark 10M Spectrophotometer (Tecan GmbH, Grodig, Austria) at 595 nm.

*Intracellular Caspase-3 and Caspase-8*

To evaluate the level of intracellular active (cleaved) human caspase-3 enzyme, a sandwich-type Elisa kit was used (from Invitrogen, through Thermo Fischer Scientific, Waltham, MA, USA) according to the manufacturer indications. This assay gives quantitative detection on the level of human caspase-3 cleaved at Asp175/Ser176 site and it is capable to recognize natural and recombinant human caspase-3. Caspase-3 was expressed as ng/mg protein.

Serial dilutions were prepared from the standard lyophilized human active caspase-3 protein, in Standard Diluent Buffer provided by the assay kit, to obtain standard concentrations between 0.039 and 2.5 ng/mL. The multiwell strips coated with anti-caspase-3 antibody were loaded with100 μL standard solutions, or cell lysate samples diluted 1:10 with Standard Diluent Buffer, in duplicates. The plate was incubated for 2 hours at room temperature to ensure the antigen binding to the wells surface, then all the strips were washed 4 times with Wash Buffer provided by the kit. 100 μL of active Caspase-3 Detection Antibody solution was dispensed into each well, incubated 1 hour at room temperature, then the wash steps were repeated. 100 μL Anti-Rabbit IgG Horse radish peroxidase solution was pipetted into each well, incubated for 30 minutes at room temperature, then the strips were washed 4 times. 100 μL Stabilized Chromogen solution was added to each sample, and the plate was kept at room temperature in the dark for about 30 minutes, and the substrate solution slowly turned blue.100 μL Stop Solution was added to each well, all samples turn into yellow, and the colorimetric assessment was performed immediately, using the Elisa plate reader, at 450/540 nm. The Magellan software outlined the standard curve, and provided the individual values; the samples being diluted 1:10, the values were multiplied accordingly.

The standard cleaved caspase-8 protein was assessed with the Human Elisa kit (Cleaved CASP8 from Mybiosource, SanDiego, CA, USA), according to the manufacturer indications. Caspase-8 was expressed as ng/mg protein.

The standard protein and the samples (cell lysates) were diluted 1:10 with Standard Dilution Buffer. The dismountable, microwell strips covered with anti-cleaved Casp8 antibody were loaded with 100 µL of standard protein solutions (7 concentrations between 7.8 and 500 pg/mL, and a blank having 0 ng/mL), or sample solutions, in duplicates. The plate was sealed and incubated for 90 minutes at room temperature, on an orbital shaker set at 100 RPM. After the incubation, the plate was discarded and 100 µL Biotin- detection antibody working solution was pipetted into each well; the plate was incubated for 1 hour at room temperature with shaking. After 3 automated wash cycles with the Wash buffer provided by the kit, 100 µL HRP-Streptavidin conjugate solution was added to each well, the plate was incubated for 30 minutes at room temperature than washed 5 times with Wash buffer. 90 µL of TMB substrate was added to all wells, and the plate was incubated at room temperature for the color development for approximately 20 minutes. 50 µL of stop solution was pipetted into each well, the plate was placed immediately into the Elisa reader and absorbance measurements were made at 450 nm. The single concentrations were calculated by the Magellan software using the standard curve, and the calculations were made accordingly to the dilution ration of the samples.

*Soluble intracellular adhesion molecule 1 (ICAM-1) and vascular cell adhesion molecule-1(VCAM-1/CD106)*

On the anti-ICAM-1 antibody coated 96 well microplate provided by the manufacturer, 100 µL standard solutions (duplicates) and samples (in triplicate) were loaded. After 90 minutes of incubation at 37 ºC, the liquid was removed from each well and 100 µL biotinylated detection antibody solution was dispensed, then the plate was incubated for 1 hour at 37 ºC. The plate was washed 3 times with the wash solution provided by the kit, using the automated washer (Plate washer from DiaSource, Louvain-la-Neuve, Belgium). 100 µL of Horse radish peroxidase conjugate solution was added to each well, and incubated for another 30 minutes. The wash procedure was repeated, the wells were incubated with 90 µL Substrate Reagent for another 15 minutes, then an acid stop solution was added to each well, and the optical density of the samples was measured with the Elisa reader (above mentioned equipment) at 450/540 nm. The single concentrations were calculated by the Magellan software using the standard curve.

The anti-VCAM-1 coated microwell strips were loaded with 100 µL of VCAM-1 recombinant protein standard solutions (from 100 to 1.56 ng/ml), sample diluent as blank, or the collected supernatant samples, in duplicates, and the plate was incubated for 90 minutes at 37ºC. The liquid was removed from the wells, without washing and in each well 100 µL biotinylated detection anti-MMP-9 antibody solution was dispensed; the plate was incubated 1 hour at 37ºC. After 3 washing cycles with the wash buffer provided by the kit, 100 µL HRP conjugate was added on the multiwall strips, and the plate was incubated 30 minutes at 37ºC. After the removal of the liquid and 3 wash cycles with the automated plate washer, 90 µL substrate reagent was added to each well, and after color development (about 15 min at 37ºC) acid stop solution was dispensed in the wells, and the plate was subjected to colorimetric absorbance measurements, at 450nm, as described above. The software of the equipment provided the single concentrations for each sample.

*Matrix metalloproteinase-9 (MMP-9)*

The anti-human MMP-9 polyclonal antibody-treated microwell strips provided by the manufacturer were washed twice, and the wells were loaded with standards (7 serial concentrations in the range of 0.23-15.00 ng/ml, in duplicates), 100 µL of assay buffer as blank, 90 µL assay buffer in each well designated for samples, than 10 µL of supernatant was added to the wells, in duplicates. To all wells 50 µL solution of Biotin-conjugate anti-MMP-9 antibody was added and the plate was placed on an orbital shaker with 100 RPM, at room temperature for 2 hours. The plate was washed four times using the automate plate washer, 100 µL of Streptavidin-Horse Radish Peroxidase solution was dispensed in the wells, and the plate was incubated on shaker for another hour. The wash cycle was repeated, and 100 µL TMB substrate solution was added on standards and samples. Within about 15 minutes, when the color development was satisfactory, acid stop solution was added to the plate, and the samples were read with the Elisa reader, as described above.

**RESULTS**

Table 5. The cell survival rate of PBMC subpopulations after 24 hours treatment with 20 μg/mL extracts from *M. aquifolium* bark (**1**), leaves (**2**), flowers (**3**), green fruits (**4**) ripe fruits (**5**); median values ± standard error of the mean (SEM) are presented for each value.

| PBMC subpopulations | Extract **1** | Extract **2** | Extract **3** | Extract **4** | Extract **5** |
| --- | --- | --- | --- | --- | --- |
| CD4+ | 65,9± 5.5 | 79.9 ± 1.8 | 94.7 ± 0.8 | 95.8 ± 0.3 | 97.0 ± 1.1 |
| CD8+ | 68.5 ± 2.9 | 87.3 ± 1.4 | 95.5 ± 2.9 | 98.9 ± 0.4 | 97.3 ± 0.8 |
| CD14+ | 77.5 ± 2.3 | 89.3 ± 1.9 | 93.6 ± 1.3 | 97.0 ± 0.7 | 95.8 ± 0.4 |
| CD19+ | 68.6 ± 1.3 | 85.8 ± 0.9 | 92.1 ± 1.7 | 90.4 ± 1.1 | 95.7 ± 1.9 |

Table 6.  Analytical parameters, concentrations and elution time related to the compounds determined on the five studied extracts.

| **Nr** | **Compounds** | **Elution time (minutes)** | **R^2^** | **LOD**  **(µg/mL)** | **LOQ**  **(µg/mL)** | **Extract 1** MB  **(µg/mL)** | **Extract 2** ML  **(µg/mL)** | **Extract 3** MF  **(µg/mL)** | **Extract 4** MRF  **(µg/mL)** | **Extract 5** MGF  **(µg/mL)** |
| --- | --- | --- | --- | --- | --- | --- | --- | --- | --- | --- |
| 1 | Chlorogenic acid | 5.41 | 0.9991 | 3.2 | 9.8 | 18.1 ±4 | 5049±25 | 2013±2 | 944±22 | 1763±7 |
| 2 | p-Coumaric acid | 9.17 | 0.9999 | 1.3 | 4.0 | 5.5±0.2 | 4.3±1.0 | 7.2±0.1 | 4.5±2.0 | <LOQ |
| 3 | Ferulic acid | 10.07 | 0.9998 | 1.4 | 4.2 | 9.2±0.0 | 11.8±1.9 | 10.0±0.3 | 7.8±0.0 | 5.6±0.2 |
| 4 | Rutin | 14.55 | 0.9996 | 2.7 | 8.1 | 268±57 | 371±18 | 73±1.6 | 26.1±0.0 | 12.9±0.0 |
| 5 | Isoquercetin | 15.34 | 0.9995 | 1.7 | 5.2 | 47.5±3.2 | 217±7.6 | 29.7±0.7 | 32.4±0.3 | 37.3±0.2 |
| 6 | Quercitrin | 24.1 | 0.9949 | 13.7 | 41.6 | <LOD | <LOD | <LOD | <LOD | <LOD |
| 7 | Berbamine | 25.5 | 0.9997 | 2.4 | 7.3 | 139.7 | <LOD | <LOD | <LOD | <LOD |
| 8 | Jatrorrhizine | 30.57 | 0.9994 | 2.7 | 8.3 | 1902.1 | <LOD | <LOD | <LOD | <LOD |
| 9 | Palmatine | 34.96 | 0.9998 | 1.7 | 5.2 | 427.9 | <LOD | <LOD | <LOD | <LOD |
| 10 | Berberine | 36.08 | 0.9996 | 2.1 | 6.4 | 1294.4 | <LOD | <LOD | <LOD | <LOD |
| *M. aquifolium*: ML - leafs, MGF – green fruits, MRF – ripe fruits, MF – flowers, MB – bark; LOD – limit of detection, LOQ – limit of quantification, R2 – curve coefficient for six levels of concentration. Indicated intervals represent average ± standard deviation (n=2). | | | | | | | | | | |

Table 7. Reference values for the total protein calibration curve:

| **μg/mL** | **Abs 1** | **Abs 2** | **Average** | **Δabs** |
| --- | --- | --- | --- | --- |
| ***0*** | 0.0998 | 0.0996 | 0.0997 | **#** |
| ***100*** | 0.4165 | 0.4144 | 0.41545 | **0.41545** |
| ***50*** | 0.354 | 0.3652 | 0.3596 | **0.2599** |
| ***25*** | 0.2718 | 0.3002 | 0.286 | **0.1863** |
| ***12.5*** | 0.2513 | 0.2615 | 0.2564 | **0.1567** |
| ***6.25*** | 0.2335 | 0.2367 | 0.2351 | **0.1354** |
| ***3.125*** | 0.2269 | 0.225 | 0.22595 | **0.12625** |
| ***1.563*** | 0.2092 | 0.2031 | 0.20615 | **0.10645** |

Figure 10 . *Mahonia aquifolium* influence on CD4+ T helper cells activation through CD25 and IL-10 pathway.

Figure 11. *Mahonia acquifolium* influence on CD8+ effector T cells activation through CD25 and IL-10 pathway.


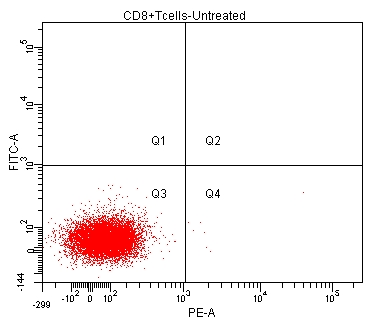

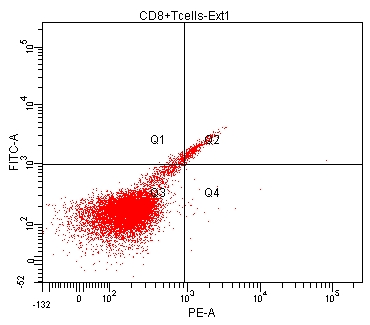

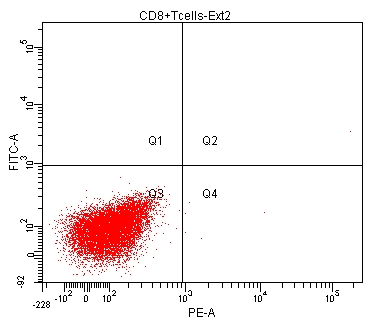

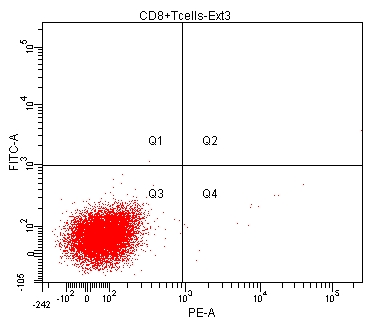

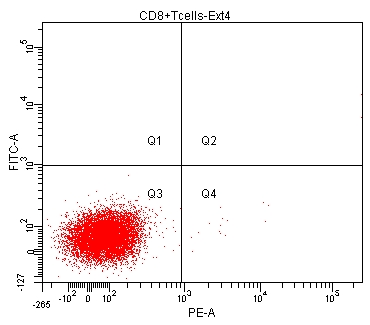

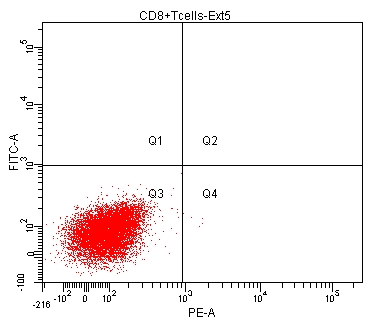

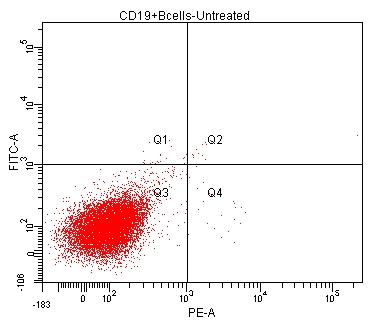

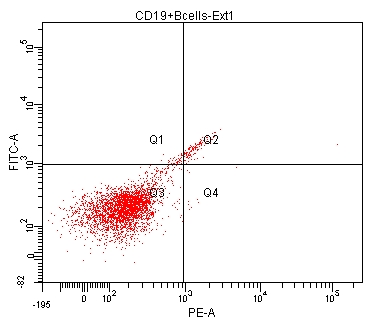

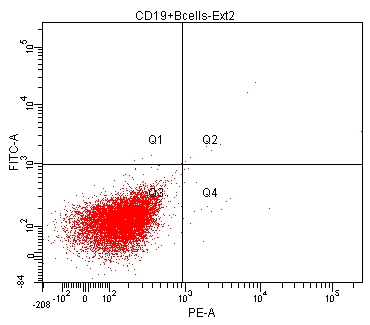

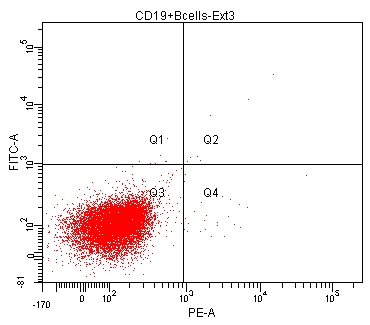

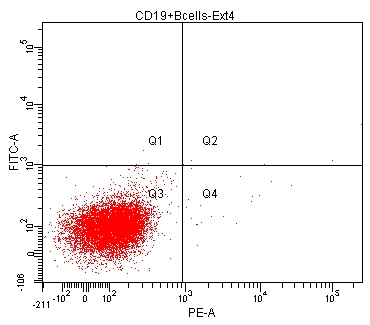

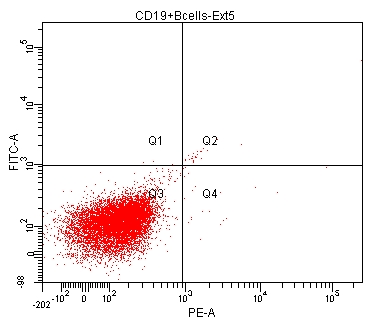


Figure 12. *Mahonia acquifolium* influence onCD19+ B cells activation through CD25 and IL-10 pathway.


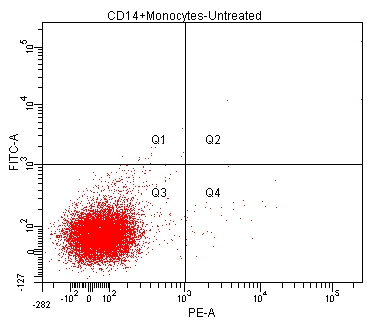

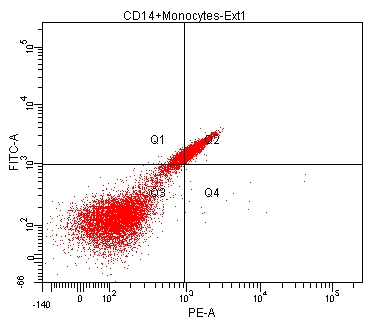

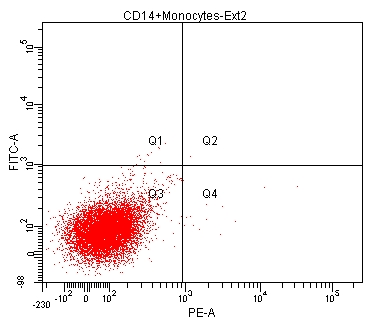

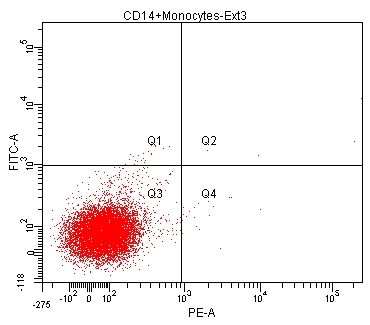

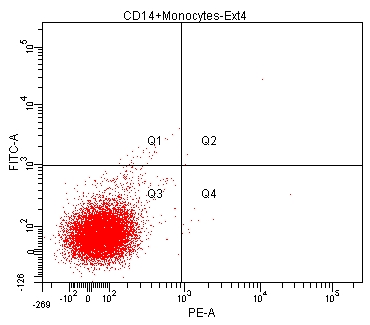

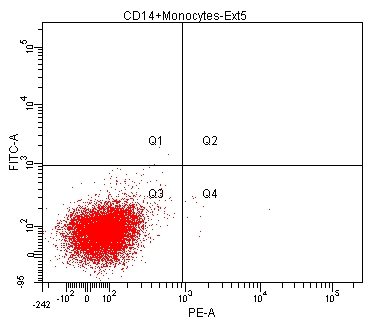


Figure 13. *Mahonia acquifolium* influence on CD14-positive monocytesactivation through CD25 and IL-10 pathway.


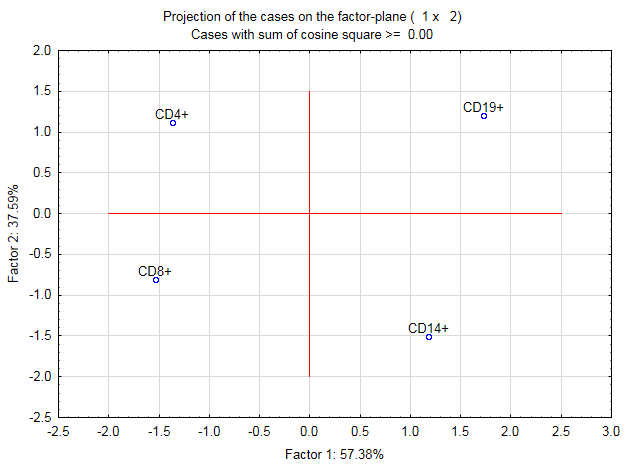


Figure 14. Score plot using the first three principal components of the five *Mahonia* extracts – PBMC subpopulations.


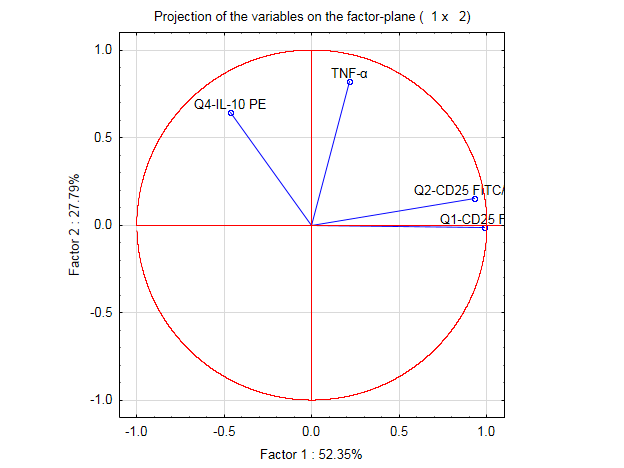


Figure 15. Correlation circle (loading plot) using the first two principal components of the PCA model obtained after applying PCA – subpopulations of lymphocytes. Considering the distances between the TNF- α secretion was correlated with the CD25^+^ and CD25^+^IL-10^+^ phenotype, but the relationship is less significant between TNF- α and the CD25^-^IL-10^+^ cells, depicted in Quadrant 4 of flow cytometry data.

.


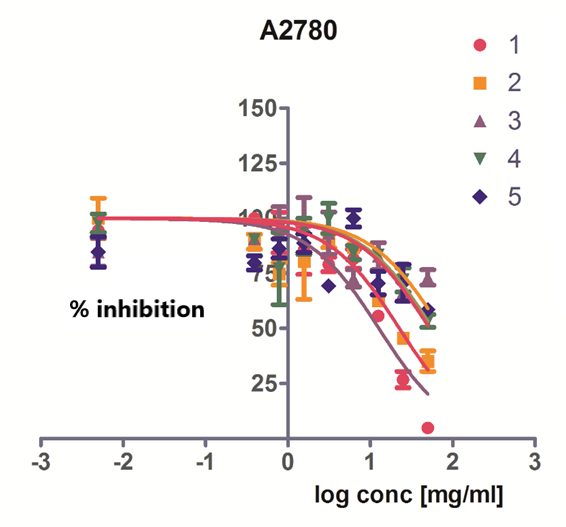

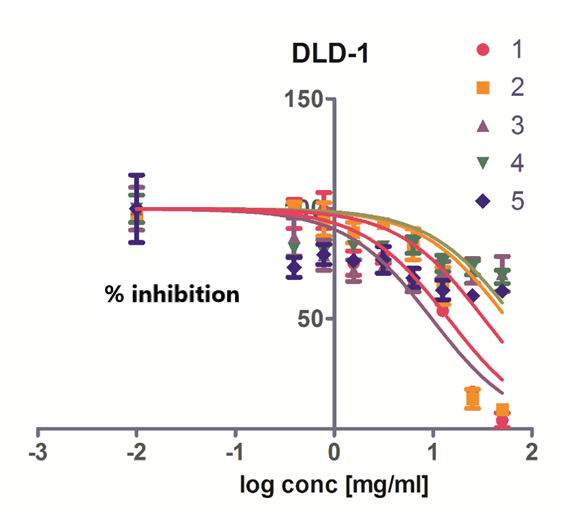

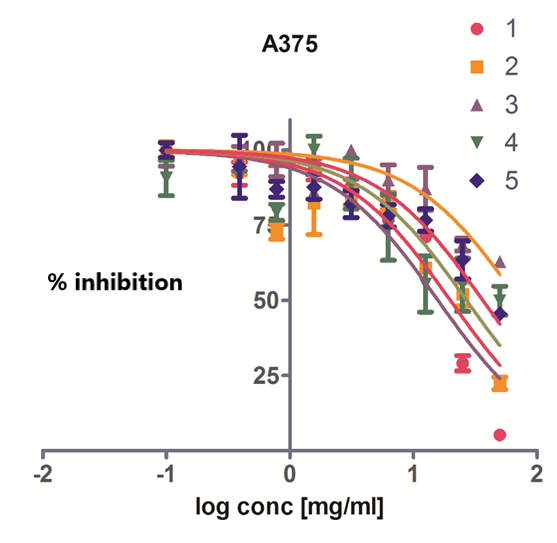

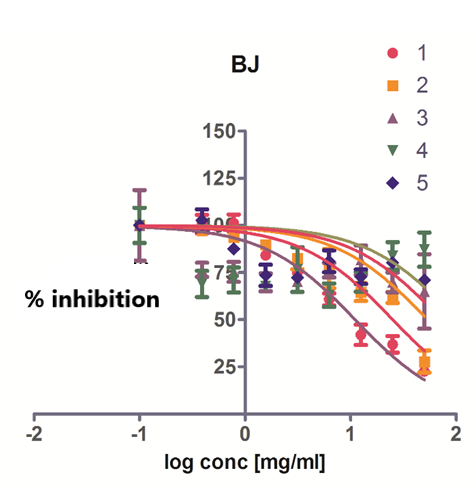


Figure 16. Sigmoidal dose-response curves depicting the relationship between the concentration of the *M. aquifolium* extracts **1**-**5** (logarithm of the concentration in μg/mL on x axis) versus the cells growth rate *in vitro*, expressed as percents on the y axis. From top down: A2780 ovary carcinoma cells, DLD-1 colon carcinoma cell lines, A375 malignant melanoma and BJ normal skin fibroblasts.


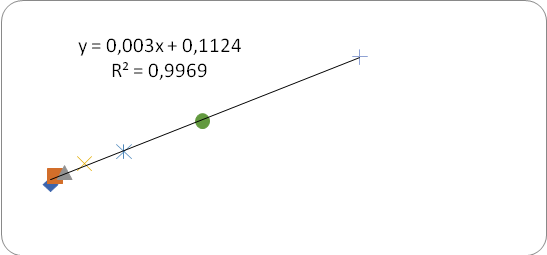


Figure 17. Calibration curve built for quantitative determination of the sample total protein content, using the Bradford method.


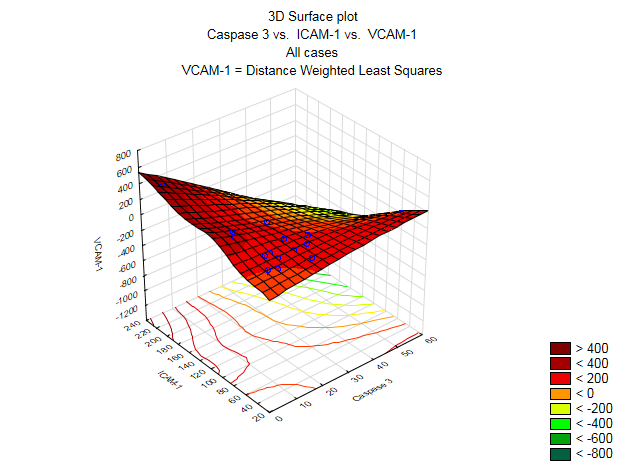


Figure 18. The relationship between the extrinsic apoptosis parameter Caspase-3 and the adhesion molecules ICAM-1 and VCAM-1, for the Mahonia extracts **1** to **5**, determined through Principal Component Analyis method.
